# Quantification of Goldmann Visual Fields During Resolution of Traumatic Optic Neuropathy

**DOI:** 10.1155/2024/5560696

**Published:** 2024-11-16

**Authors:** Midori Tachibana, Junji Kanno, Miho Hashimoto, Yu Hosokawa, Masafumi Sawada, Yuri Nishiyama-Ota, Satomi Konno, Rintaro Aoyagi, Sho Ishikawa, Jun Makita, Tetsuo Ikezono, Kei Shinoda

**Affiliations:** ^1^Department of Ophthalmology, Faculty of Medicine, Saitama Medical University, Saitama, Japan, 38 Moro-Hongo Moroyama-machi, Iruma-gun, Saitama 350-0495, Japan; ^2^Department of Otorhinolaryngology, Faculty of Medicine, Saitama Medical University, Saitama, Japan; 38 Moro-Hongo Moroyama-machi, Iruma-gun, Saitama 350-0495, Japan

**Keywords:** critical fusion flicker, Goldmann visual field, quantitative evaluation, traumatic optic neuropathy, visual field

## Abstract

**Purpose:** We describe the clinical findings in a Japanese patient with unilateral traumatic optic neuropathy (TON) who underwent steroid pulse therapy followed by optic canal decompression surgery. The optic nerve function was assessed longitudinally and quantitatively by Goldmann visual fields (GVFs). This was accomplished by measuring the area of each isopter and scotoma, and the findings were compared with the visual acuities recorded during the course of the resolution of the TON.

**Case Presentation:** A 70-year-old man suffered from TON in his left eye after falling from a 3 m cliff. On examination at the Saitama Medical University Hospital, his decimal best corrected visual acuity (BCVA) in his left eye was 0.2. Computed tomography revealed a fracture of the lateral wall of the left orbit, but no obvious optic canal fracture was observed. Steroid pulse therapy was started, and optic nerve decompression surgery was performed. After steroid administration, there was a slight improvement in the visual acuity, and a response in the I/3e isopter was present (area 17.76 deg^2^). However, the V/4e area decreased from 539.35 to 359.36 deg^2^. Three days after the optic canal decompression surgery, the decimal visual acuity improved to 0.6, and the V/4e area and I/3e area increased to 1122.52 and 46.88 deg^2^, respectively. Postoperatively, there was a marked improvement in the size of the GVFs that corresponded to the improved visual acuity. The visual acuity of the left eye was 0.8 after 6 months, and the GVF was still not completely normal.

**Conclusions:** Our findings showed the course of recovery of the visual acuity and visual field in an eye with TON. The quantification of GVF was helpful in assessing the course of recovery after the treatments. The new quantitative index of the GVFs may be helpful in evaluating the effectiveness of treatments for optic nerve disorders.

## 1. Background

Traumatic optic neuropathy (TON) is caused by an acute optic nerve injury from a direct or indirect trauma that results in a reduction of visual acuity. The severity of the optic nerve damage varies from a simple contusion to a complete avulsion of the optic nerve. Indirect damage to the optic nerve can also cause TON which occurs in 0.5%–5% of all closed-head traumas [1, 2]. Although the degree of visual acuity reduction in indirect TON varies, one-half of the cases have visual acuities of light perception or even no light perception [3–5].

No established treatment exists for TON, and its optimal management protocol is under debate [3–7]. The International Optic Nerve Trauma Study (IONTS) group reported that there were no significant differences in the visual outcomes between those treated with surgical decompression and those treated with steroids. Thus, neither corticosteroid therapy nor optic canal decompression is the better treatment of TON [6].

We present a case of TON that underwent steroid pulse therapy, followed by optic canal and nerve sheath decompression surgery. The optic nerve function was assessed by Goldmann visual fields (GVFs). The GVFs were determined multiple times because the defects extended beyond the central 30°. The GVF results were analyzed quantitatively and compared with the best corrected visual acuity (BCVA) and the critical fusion flicker (CFF) frequency during the course of the disease process.

## 2. Case Presentation

A 70-year-old man fell from a 3 m cliff and landed on the left side of his face. Six days later, he visited an eye clinic complaining of blurred vision, and he was referred to the Saitama Medical University Hospital with a diagnosis of TON. Our examination showed that his decimal BCVA was 0.9 in the right eye and 0.2 in the left eye. A relative afferent pupillary defect was detected in the left eye, and the eye movements were normal bilaterally. Fundoscopy revealed a healthy-looking optic disc with normal retinal circulation bilaterally. The intraocular pressure was 10.0 mmHg in both eyes. The CFF was 37.0 Hz in the right eye and unmeasurable in the left eye.

**Figure 1:**
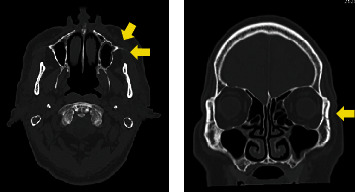
Computed tomographic images at the initial visit of a 70-year-old man with traumatic optic neuropathy (TON). Axial and coronal computed tomographic images show a fracture in the lateral wall of the left orbit. The anterior and posterior walls of the left maxillary sinus are fractured and slightly depressed. No obvious optic canal fracture can be seen.

Computed tomographic examination revealed a fracture in the lateral wall of the left orbit (Figure 1), and the anterior and posterior walls of the left maxillary sinus were fractured and slightly depressed. No obvious optic canal fracture was seen.

**Figure 2:**
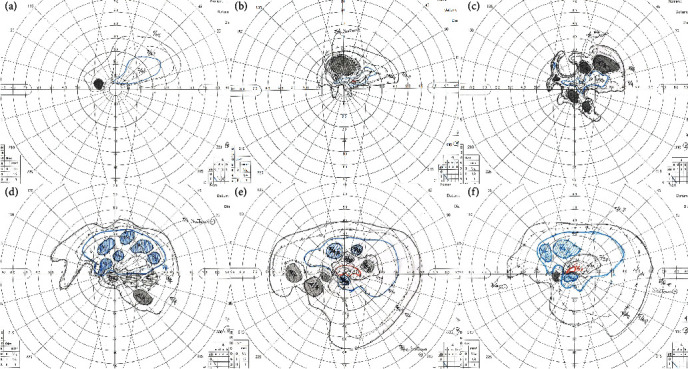
Serial tests of Goldmann visual fields throughout the disease course. (a) The decimal visual acuity at the first visit was 0.2, and there was no response to the 1/3e isopter and larger isopters. (b) On the second day of steroid pulse therapy, there is a slight improvement in the visual acuity and response in the I/3e isopter, but the responses to V/4e, III/4e, and I/4e have decreased. (c) On the 11th day after the injury, the V/4e isopter extends downward, but there was no change in the visual acuity. The upper visual field of the V/4e isopter is collapsed, and the absolute scotoma has expanded to the center. (d) On the 3rd day after the surgery, the visual acuity is 0.6, and the scattered absolute scotoma becomes a relative scotoma in the upper visual field, and improvements can be seen. (e) Six weeks after the surgery, the decimal visual acuity was 0.7. The lower visual field improved despite the residual paracentral scotoma, and responses for I/2e and I/1e at center 5°–10° are present. (f) Six months after the surgery, the visual acuity was 0.8. Compared with 6 weeks after surgery, the lower V/4e isopter appears to be slightly smaller. The blue line indicates I/4e isopter, and the red line indicates I/2e isopter.

**Figure 3:**
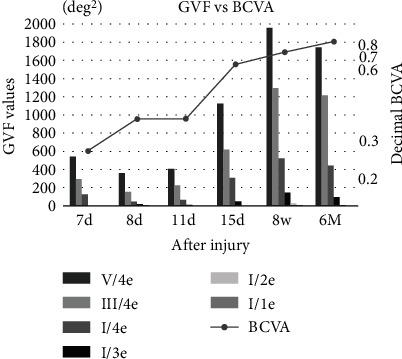
Changes of visual acuity and the area of each isopter for the Goldmann visual fields throughout the course of the disease process. The figure is combined with a table showing the absolute value of the area and its increased ratio over the previous value. The initial visual acuity was 0.2, and Goldmann visual fields (GVF) showed no response in the I/3e or larger isopters (area 0 deg^2^). After steroid administration, there was a slight improvement in visual acuity, and a response in the I/3e isopter was preset (area 17.76 deg^2^). However, the V/4e area decreased from 539.35 to 359.36 deg^2^. Three days after the optic canal decompression surgery, the visual acuity improved to 0.6, and the V/4e area and I/3e area increased to 1122.52 and 46.88 deg^2^, respectively. The critical fusion flicker (CFF) frequency became measurable at 16.3 Hz in the left eye. Eight weeks after the surgery, the visual acuity was 0.7; although a paracentral relative scotoma remained, improvements of the V/4e area to 1955.79 deg^2^ and the I/3e area to 114.63 deg^2^ were observed. The CFF frequency was 17.0 Hz. Six months after the surgery, the visual acuity further improved to 0.8, the CFF frequency was 24.7 Hz, and the V/4e area and I/3e area decreased to 1744.14 and 95.36 deg^2^, respectively.

Then, 1 g/day of intravenous methylprednisolone was started. Two days later, the visual acuity in the left eye improved to 0.3, but the GVFs had worsened (Figure 2). The GVF examination showed an area of 17.76 deg^2^ for the I/3e isopter (Figure 3). However, the V/4e area decreased from 539.35 to 359.36 deg^2^.

The patient then underwent optic nerve decompression by an endoscopic endonasal approach, and a bone fragment was removed from the optic canal. Three days after the surgery, the decimal BCVA improved to 0.6, and the V/4e area and I/3e area enlarged to 1122.52 and 46.88 deg^2^, respectively. The CFF frequency became measurable at 16.3 Hz in the left eye. Eight weeks after the surgery, the visual acuity was 0.7, although a paracentral relative scotoma was still present. Improvements of the V/4e area to 1955.79 deg^2^ and the I/3e area to 114.63 deg^2^ were detected. The CFF frequency was 17.0 Hz in the left eye. Six months after the surgery, the visual acuity further improved to 0.8, and the CFF frequency was 24.7 Hz in the left eye. However, the V/4e area and I/3e area decreased to 1744.14 and 95.36 deg^2^, which were 0.85 and 0.66, respectively, of the previous values. The changes in the BCVA and the area of each isopter of the GVF are shown in Figures 2 and 3.

### 2.1. Quantitative Analyses of GVFs

The GVFs were quantified with the ImageJ (https://imagej.nih.gov/ij, accessed on 31 May 2023) software [8–11]. For this, we constructed an ImageJ macro called the Kanno–Saitama Macro (KSM)-GVF program by modifying the already existing KSM program whose details have been reported [12, 13]. In brief, the KSM-GVF program consisted of two macros, one for the extraction and one for the quantification of the size of the visual fields for each isopter. Continuous quantification was also possible by rewriting the macro code. The extraction followed by quantitation is sequentially shown. The procedures are shown as follows:

A. A black magic marker was used to trace each isopter and its scotoma on two copies of papers of the GVF results. One copy was for each isopter, and the other was for the scotoma. The two traced sheets were scanned and imported to ImageJ, and the macro for extraction was started.
B. For isopter extraction, the auto threshold minimum macro [14] in ImageJ was used. When the macro was activated, the size inside the traced black magic marker was calculated and extracted.
C. After each isopter was extracted, the number of pixels in the area surrounded by the isopters was quantified by activating the macro for quantification. Next, the number of pixels within each isopter was divided by the number of pixels in a 15 × 15 deg^2^ area of the same image. Finally, the value was multiplied by 225 (15 squared) to obtain the area (deg^2^) of each isopter and each scotoma.

The results of the quantification showed that the size of the GVF increased and that of the scotoma decreased with increasing posttreatment times (Figure 2).

## 3. Discussion

We have presented our findings in a case of TON in which the visual functions were quantitatively monitored during and after steroid pulse therapy combined with optic nerve decompression surgery. Our findings showed that there were improvements in both the BCVA and the GVFs, and the improvements were corresponding.

Clinical interventions for TON include observation only, megadose steroid administration, and/or optic canal decompression with or without steroids. The best treatment has still not been definitively determined. A review of the earlier studies showed that the visual acuity of patients with TON can be significantly improved after optic canal decompression surgery, independent of steroid use [7]. The rationale for surgical decompression is that it can remove the structures compressing the optic canal and thus remove the detrimental effects of compression. The decompression lowers the intracanalicular pressure and enables the removal of any impinging bone fragments, allowing nerve function to be restored. Systematic steroids have a similar effect, leading to a medical decompression [15].

Static perimetry is used more widely than kinetic perimetry today in ophthalmic practice. However, kinetic perimetry remains helpful in evaluating the progression of inherited, autoimmune retinal degenerations, and severe optic neuropathy, where the visual field defects or scotomas lie beyond the central 30° of the visual field [16]. Descriptive methods for assessing the GVFs have been used in clinical settings. In contrast, several methods have been proposed to quantify the GVFs such as the use of planimetry [17–19], the use of the American Medical Association (AMA) score [20], and digital quantification using Adobe Photoshop [21, 22].

In our case, we have used the KSM macro with ImageJ to obtain quantitative values of the size of the GVFs. Thus, the course of the disease process can be easily determined by the changes in the quantified data. We found that the BCVA improved slightly, but the GVFs improved markedly after the steroid pulse therapy, especially after the decompression surgery. The quantification of the size of the VDs showed that the size of the GFVs I/3e isopter had the greatest increase from the earlier value, suggesting that there is a correlation between the recovery of central visual fields and the improvements of visual acuity. At 6 months after the surgery, the BCVA was still improving, but the GVF values started to decrease. Although the influence of fluctuations inherent in subjective tests cannot be denied, it is possible that the improvements in the BCVA and the GVF had passed their peak. This clearly indicated that functional decline can occur even after a significant recovery due to the treatments. This suggests the importance of obtaining quantitative indices of the visual field function in clinical practice.

The KSM-GVF can measure the area of each isopter and scotomata in a time-saving, reproducible, and reliable manner. It can follow the longitudinal changes in the GVF quantitatively and accurately. This will then allow clinicians to monitor the progress of the disease process and determine the effectiveness of the therapy being administered.

There are several limitations in this study. First, because we have reviewed the patients' medical records, there was a lack of structural parameters such as retinal nerve fiber layer thickness or retinal capillary density in the superficial retinal layer. Both findings are important because they have been reported to reflect the retinal ganglion cell damage [23, 24]. Second, the KSM-GVF program calculates only the area of each isopter and does not consider differences of sensitivity within the VF; for example, the central part of the isopter is more sensitive than the peripheral part. Therefore, it may be better to sum the KSM-GVF indices on a weighted scale to measure the volume corresponding to each isopter. This would consider the sensitivity and area into account. For this limitation, Odaka et al. proposed a system that can digitize the isopters to calculate the area, volume, and shape factors [25]. Third, the findings are from a single case and cannot be generalized. However, no new equipment is required, and analysis can be performed on the results already obtained using the widely used GFVs. New quantitative metrics may be useful in the interpretation of the visual fields.

## 4. Conclusion

We have presented the functional changes caused by a unilateral TON in a 70-year-old Japanese man using quantitative analyses of the GVFs. Although the data were from a single case and cannot be generalized, the findings showed a significant improvement of the GVFs that corresponded with the improvements of the BCVA after the optic canal decompression. Additional cases and further quantitative analyses of the GVFs should provide new insights into the usefulness of multimodal functional evaluations of optic nerve disorders.

## Data Availability

All data generated or analyzed during the current study are included in this published article.
